# Reference Human Rotavirus A Genome Sequence from a Previously Vaccinated Child with Diarrhea in Nigeria

**DOI:** 10.1128/MRA.01352-19

**Published:** 2020-01-30

**Authors:** T. O. C. Faleye, U. E. George, C. Simsek, O. A. Arowolo, O. M. Adewumi, J. Matthijnssens, J. A. Adeniji

**Affiliations:** a Centre for Human Virology and Genomics, Department of Microbiology, Nigerian Institute of Medical Research, Lagos, Nigeria; b Department of Virology, Faculty of Basic Medical Sciences, College of Medicine, University of Ibadan, Ibadan, Nigeria; c Department of Biological Sciences, Redeemer's University, Ede, Nigeria; d KU Leuven, Department of Microbiology, Immunology and Transplantation, Rega Institute, Laboratory of Viral Metagenomics, Leuven, Belgium; e Department of Veterinary Microbiology, National Veterinary Research Institute, Vom, Nigeria; f Infectious Disease Institute, University of Ibadan, Ibadan, Nigeria; g WHO National Polio Laboratory, University of Ibadan, Ibadan, Nigeria; Portland State University

## Abstract

In 2018, a 26-month-old girl, fully vaccinated with Rotarix in 2016, presented with fever, diarrhea, and vomiting. A rapid test showed that her feces contained rotavirus A (RVA). VP7 reverse transcription-PCR (RT-PCR) and Illumina sequencing showed that a G1P[8] strain with a Wa-like genotype constellation was the etiologic agent. This is the first near-complete RVA genome sequence from Nigeria.

## ANNOUNCEMENT

Rotavirus A (RVA) belongs to the genus Rotavirus, family Reoviridae. The virus has a double-stranded RNA (dsRNA) genome with 11 segments (1). It is the most important etiologic agent globally of diarrhea in children under 5 years of age (2). Two globally licensed vaccines (Rotarix and RotaTeq) have been developed for the control of human RVA (3). In Nigeria, RVA vaccination is recommended for children as part of the routine immunization program. However, it is currently only available for a fee. Consequently, only those who can afford to pay for the vaccine have it administered to their infants. Here, we describe the first near-complete genome of a wild-type RVA strain from Nigeria from a child who had been previously vaccinated with Rotarix.

A fecal sample was collected in 2018 from a 26-month-old girl who presented with fever, diarrhea, and vomiting. The sample was subjected to a rapid immunochromatographic test for both RVA and enteric adenoviruses (rotavirus group A antigen/enteric adenovirus antigen rapid test kit [AccuMed Technology Co., Ltd., Beijing, China]). Subsequently, RNA was extracted, followed by cDNA synthesis, VP7 nested multiplex PCR, and gel electrophoresis (4). Afterwards, the sample was subjected to the novel enrichment technique of the VIRomes (NetoVIR) protocol (5). Briefly, the sample was homogenized, centrifuged for 3 min at 17,000 × *g*, and filtered using a 0.8-μm centrifugal filter. The filtrate was then treated with nucleases (benzonase and microccocal) and subsequently subjected to RNA extraction, cDNA synthesis, and full-genome amplification using the WTA2 kit, library preparation using the Nextera XT DNA kit, and Illumina sequencing. Paired-end (2 × 150-bp) sequencing was done using the NextSeq platform (Illumina). Trimming and assembly were done using Trimmomatic v1.2.14 (6) and SPAdes v1.2.3 (7), respectively. DIAMOND v1 was used to annotate the obtained contigs (8). All software was used with default settings.

The rapid test showed that the sample contained RVA. The VP7 assay confirmed this and typed it as G1. The NetoVIR metagenomics protocol yielded 7,698,832 reads after quality trimming. Eleven near-complete genome segments of an RVA strain were recovered (Table 1) from 6,136,952 (79.71%) of the reads. This strain was typed as G1P[8] with a Wa-like genotype constellation (9), using the RotaC classification tool (10). All 11 segments showed a range of 98.9% to 99.9% similarity with known contemporary RVA strains (Table 1). Precisely 6.2% (48/777) and 5.2% (17/326) of the amino acid residues were different between the VP4 and VP7 proteins of the Nigerian G1P[8] strain reported here and the Rotarix vaccine strain.

**TABLE 1:** Assembly and genotyping details of the RVA genome described in this study

Segment no.	Segment length (bp)	Coverage (×)	No. (%) of mapped reads	GC content (%)	ORF^*a*^ length (bp) (protein product)	Genotype	Closest strain in GenBank	Similarity (%)
1	3,300	45,979	1,265,688 (16.44)	32.7	1,088 (VP1)	R1	JQ069935	99.2
2	2,755	33,210	770,838 (10.01)	31.7	894 (VP2)	C1	MN066755	99.7
3	2,589	62,609	1,376,287 (17.88)	30.0	835 (VP3)	M1	LC469459	99.0
4	2,340	47,186	917,626 (11.92)	33.3	777 (VP4)	P[8]	MG652340	99.4
5	1,563	61,422	824,708 (10.71)	31.1	493 (NSP1)	A1	LC374132	99.1
6	1,345	2,284	27,146 (5.68)	37.9	397 (VP6)	I1	LC469482	99.9
7	1,060	50,245	441,890 (5.74)	34.2	326 (VP7)	G1	KX638552	98.9
8	1,059	10,262	89,639 (1.16)	31.1	317 (NSP2)	N1	MK302413	99.6
9	1,058	38,700	335,057 (4.35)	30.3	310 (NSP3)	T1	KU738580	99.5
10	757	11,314	70,732 (0.92)	32.9	175 (NSP4)	E1	EU679378	99.0
11	668	3,130	17,341 (0.23)	39.3	197 (NSP5)	H1	MG181479	99.4
					92 (NSP6)			

a All open reading frames (ORFs) were complete.

We describe here what is, to the best of our knowledge, the first near-complete human RVA genome sequence from Nigeria. This genome will serve as a reference for the region and form part of the baseline data needed to address the outstanding issues of rotavirus vaccine failure in Nigeria and globally (10).

### Data availability.

The assembled genomes have been deposited in GenBank under the accession numbers MN304722 to MN304732. The raw reads have been deposited under SRA accession number SRX7053324.
